# International Perspectives on Mental Health Social Work: Second Edition

**DOI:** 10.3390/ijerph21030336

**Published:** 2024-03-13

**Authors:** Jim Campbell, Lisa Brophy, Gavin Davidson

**Affiliations:** 1 School of Social Policy, Social Work and Social Justice, University College Dublin, D04 V1W8 Dublin, Ireland; 2 Social Work and Social Policy, La Trobe University, Melbourne, VIC 3086, Australia; 3 School of Social Sciences, Education and Social Work, Queen’s University Belfast, Belfast BT7 1NN, UK

Writing this Editorial for our second collection of papers on “International Perspectives on Mental Health Social Work”, we reflected upon the content of our First Edition [1]. In this edition, we sought to highlight a range of selected themes that we felt were of relevance to law, policy making, and theoretical perspectives that can inform mental health social work practice. This included a discussion about the origins of its role, with its foundations in Victorian philanthropy, and the development of social workers alongside other professionals in the 20th century [2]. In our First Edition, we also drew attention to the evolving, often problematic, interface between practitioners and mental health service users; the importance of both context and universal human rights; and the balance between individual and more community-level practice. It is not a surprise that in this work we include other papers that further explore our understanding of these crucial aspects of policy and practice. 

The first paper of the Second Edition [Contribution 1] explores the complex and evolving nature of mental health social work’s relationship with the law, in a range of national and international settings. The United Nations Convention on the Rights of Persons with Disabilities (CRPD) is a growing presence in the development of mental health and capacity laws across the world [3]. Therefore, we are pleased to report a comparative European study that was designed to capture the views of key individuals regarding the implications for the role of mental health social work in various jurisdictions. The study begins by exploring the literature on the topic, highlighting the wide-ranging aims and intentions of the convention and the often-controversial implications for policy and law makers. Four findings emerged from their rigorous literature review, including the impact upon legislation, positive and negative effects on practice, as well as implications for social work education and research. These findings help prepare the ground for future research and consideration of these significant interfaces. 

The next paper in this collection [Contribution 2] is a meticulous review of the experiences of mental health social workers across varied national, policy, and inter-professional contexts. In this review, 2132 papers were initially identified and 35 were chosen that met the inclusion criteria. Three key themes emerged from their analysis, which in many ways reinforce the findings from papers already discussed in this Editorial. Mental health social workers often agreed that they occupied a unique role in dealing with the social contexts of mental distress and issues of risk [4], and in using systemic approaches to recovery. In some legal situations, they were also found to provide legal advice and guidance, but, as is discussed in other research, they were concerned about how the mental health social work role could be marginalised in some organisational structures. These findings, as with others discussed above, highlight the need to further research the nature of the knowledge and skills that are being used and advocated by the profession in institutions. 

As mentioned previously in the First Special Edition, the important issue of service user empowerment in the process of carrying out mental health social work is of considerable interest across mental health systems [5,6]. We are, therefore, pleased that three papers on this topic are now presented in our Second Special Edition. The next paper [Contribution 3] traces the history of ideas of service user empowerment that emerged in the late 20th century with, at least in some countries, a commitment from the state to ensure the engagement of public welfare organizations with these approaches. In an argument that is often rehearsed in discussions about the changing nature of welfare regimes in the 21st century, the author describes and analyses the way in which the concept of service user empowerment is now more likely to be informed by market and individualistic assumptions. This position is then explored through an ethnographic study in Sweden, which revealed a number of personal narratives and experiences of mental health issues suggesting that there is commodification of these services, reducing opportunities for service users to challenge approaches and act independently of the state and public welfare services.

Such ideas and findings have relevance in other countries and contexts. In the next paper, the authors [Contribution 4] also challenge conventional narratives and practices of service user empowerment, this time through an Irish lens, and through interrogating notions of recovery. As with Erikson, they critically analyse the history of the idea and ways in which recovery are understood and represented in mental health systems. In this study, the position of the Irish mental health social worker is discussed and situated in the wider international literature. They report on a qualitative, stakeholder study that involved professionals, service users, family members, and policy influencers. They argue for a more central role for mental health social workers, one that moves beyond the traditional notion of recovery and enables an appreciation of the intersectional injustices and inequalities faced by hard-to-reach populations. To combat the limitations of more individual-focused practice, the authors offer some suggestions for changes to the mental health social work role which have pertinence for the profession, both locally and internationally.

A third paper on this topic examines how new technologies [7] are used to achieve forms of service user empowerment, this time in an Australian setting. The study [Contribution 5] reports on a project in a forensic mental health facility where a digital bytes project sought to capture the lives, aspirations, and perspectives of the facility’s service users. Unusually, it was co-designed and co-delivered with social work students and service users, focusing on services users’ experiences of stigma, mental distress, and the justice system. It was hoped that the findings would have implications for staff and service delivery. In conducting this study, the authors revealed the concept and use of the digital byte as a way of evaluating and changing organisational approaches to forensic mental health services. Looking to the future, where new technologies are sure to shape the delivery of these and other services, it is imperative for mental health social workers to embrace these changes. 

In the conclusion of this Second Special Issue, we summarise two papers that focused on the role of mental health social work, but outside of Western paradigms. This was one of our ambitions for both Special Issues, to rebalance colonial and neo-colonial assumptions about policy, practice, and organisational delivery [8]. The first examines the case of Guyana [Contribution 6] and sets the scene for discussion of the complex nature of the mental health social worker role. As with other papers in this collection, it pays attention to the past, in this case a colonial past characterised by economic instability, racial polarisation, and physical and mental trauma. As in other post-colonial societies, the past lingers ominously within the present. As a result, there are weaknesses and failures within mental health systems and high levels of mental ill health and rates of suicide, and these issues are associated with socio-economic inequalities and violence. Other aspects of Guyanese society also emerge in this account, including issues of religion and the presence of traditional supernatural healers and remedies used to support citizens with mental health problems. In this context, the authors introduce to the reader the concept of ‘Obeah’. One of the key features of the paper is a willingness to examine the importance of critical theory, in terms of Marxist and feminist ideas, to reframe and challenge conventional biologically based mental health paradigms. Social workers, they argue (as other papers above have), are well positioned to occupy important professional spaces, working collaboratively with other health-related professions to embrace human rights and social justice approaches that can combat the colonial past and look to the future. 

Our final paper in this collection also explores different, alternative forms of social work interventions, this time with clients with Autism Spectrum Disorder (ASD) in Ethiopia. The authors [Contribution 7] describe a qualitative study involving interviews with 20 mothers. Their findings revealed painful and poignant experiences, including issues of grieving associated with their child’s diagnosis and its emotional impact; how mothers have found ways of understanding and defining the diagnosis and their experience of autism; and then reports of finding coping strategies to raise their children. Using a feminist analysis, the authors bring to light the life and experiences of the mothers, in their journey of parenting and mothering and their new mothering roles. It is argued that the findings have resonance for the development and delivery of services for Ethiopian social workers and for those engaging with the Ethiopian diaspora in other regions of the world.

## Conclusions

This valuable collection of papers from across the globe confirms that social workers employed across the field of mental health continue to focus on fundamental professional values and human rights principles. However, the efforts of practitioners to maintain a distinct professional role and identity can be hindered by organisational pressures. Yet, there are encouraging findings in these papers regarding pathways for mental health social workers to negotiate complexities, and sometimes contradictory expectations, whilst sustaining a commitment to the needs and goals of service users. Innovative engagement with people with lived experience, valuing service users’ expertise, and adopting empowering research methods such as co-design and co-production should characterise contemporary mental health social work practice. In doing so, these approaches have the potential to support the long-standing need to transform mental health services. This implies a shift away from a risk-averse and biomedically dominated system to one that is people centred and recovery oriented, and which acknowledges the role of social determinants and inequality in mental health services. Finally, we wish to reaffirm the imperative to elevate our understanding beyond Western paradigms and in doing so enable mental health social workers to embrace and engage with critical international perspectives [9].
